# Maternal cytomegalovirus immune status and hearing loss outcomes in congenital cytomegalovirus-infected offspring

**DOI:** 10.1371/journal.pone.0240172

**Published:** 2020-10-09

**Authors:** Gail J. Demmler-Harrison, Jerry A. Miller, On behalf of the Houston Congenital Cytomegalovirus Longitudinal Study Group

**Affiliations:** 1 Baylor College of Medicine, Houston, Texas, United States America; 2 Department of Pediatrics, Section of Infectious Disease, Texas Children’s Hospital, Houston, Texas, United States of America; Texas A&M University College Station, UNITED STATES

## Abstract

**Objectives:**

The purpose of this study is to determine the relationship between maternal primary and recurrent CMV infection during pregnancy, symptoms at birth in the newborn, and long term hearing loss through18 years of age.

**Patients and methods:**

237 mother-infant pairs in the Houston, Texas area identified through maternal CMV IgG and IgM antibody serologic screening and newborn screening using urine CMV culture to identify congenital CMV infection were enrolled in the Houston Congenital CMV Longitudinal Study. Mothers were categorized as having primary or recurrent or unknown maternal CMV infections, and newborns were categorized at birth as having symptomatic or asymptomatic congenital CMV infection, or as uninfected controls. All three newborn groups were followed longitudinally with serial hearing evaluations up to 18 years of age. The relationship between type of maternal CMV infection, newborn classification, and the occurrence of hearing loss over time was determined through Kaplan-Meier survival analysis, life table analysis, and a simulated ascertainment of maternal infection type for the unknown categories.

**Results:**

Of 77 newborns with symptomatic congenital CMV infection, 12 (16%) of mothers had a primary CMV infection during pregnancy; 4 (5%) had a non-primary infection, and the type of infection in 48 (79%) could not be determined and were classified as unknown type of maternal infection. Fifty Seven (74%) of the 77 symptomatic children had hearing loss by 18 years of age, including 9 of the 12 (75%) who were born to mothers with primary infection and 48 (79%) of the 61 with unknown type of maternal infection. Of the 109 newborns with asymptomatic congenital CMV infection, 51 (47%) were born to mothers with a primary CMV infection during pregnancy, 18 (17%) to mothers with a recurrent infection; and 40 (37%) had unknown type of infection. Of these 109 asymptomatic cases, 22 (20%) developed hearing loss, including 14 out of 51 (28%) of those born to mothers with primary infection, two out of the 18 (11%) born to mothers with recurrent infection, and 6 out of the 40 (15%) to mothers of unknown infection type. Of the 51 uninfected newborn controls, 10 (20%) of mothers had a primary CMV infection during pregnancy, 5 (10%) had a non-primary infection, 10 (20%) were never infected, and 26 (51%) were assigned unknown type of infection. Three controls (6%) developed hearing loss, with 1 being born to a mother with primary infection and 1 to a mother never infected with CMV.

**Conclusions:**

Both primary and non-primary maternal CMV infections during pregnancy resulted in symptomatic and asymptomatic congenital CMV infection. Symptomatic congenital CMV infection was more likely to occur after primary maternal CMV infection. Sensorineural hearing loss occurred in children born to mothers with both primary and non-primary CMV infections, and in both asymptomatic and symptomatic congenital CMV infection, but was more common after maternal primary infection. Most, but not all, hearing loss in children with cCMV associated hearing loss was first detected within the first year of life.

## Introduction

Maternal infection with cytomegalovirus (CMV) resulting in congenital CMV (cCMV) infection continues to be a serious public health problem, affecting cCMV-infected fetuses, newborns and children. Congenital cytomegalovirus infection is a common congenital infection, with an estimated birth prevalence of 1%, ranging from 0.2% to 2.5%, depending on the population studied, resulting in tens of thousands of newborns each year born with cCMV infection in the United States [1].

The various health outcomes of congenital CMV infection may be apparent in the fetus and newborn, with one or more symptoms at birth; or become apparent later in infancy, childhood or adolescence in an otherwise asymptomatic, normal-appearing newborn. It is estimated that 10 to 15% of newborns with cCMV infection will be symptomatic at birth, with significant risk of sensory, neurological and motor sequelae [2]. The majority (85–90%) of newborns with cCMV infection will appear asymptomatic at birth, yet roughly 20% of these will have congenital or late-onset sensorineural hearing loss [3]. Hearing loss, widely accepted as being the most common sequelae of congenital CMV infection, may also first develop later in life, in both symptomatic and asymptomatic newborns, and may progress in severity once it is detected [2, 3].

Maternal infection with CMV may be from a primary infection (first experience with the virus) or a non-primary, recurrent infection (or second experience with the virus) [4–6]. Recurrent infection may be from a reactivation of the mother’s own endogenous strain or strains, or genotypes, of CMV, or from a reinfection with a new “outside’ strain or genotype of the virus. The timing of maternal CMV infections may be estimated according to trimester or gestational age of the pregnancy for primary infections, but establishing the time of recurrent CMV infections is not yet possible with certainty with available serologic or biologic markers, or other laboratory techniques.

Conflicting opinions exist about the influence of the type and timing of maternal CMV infection during pregnancy and the outcome in the child. Most studies stress the importance of primary maternal CMV infections, especially those occurring early in pregnancy, as having the greatest impact on the outcome of the newborn and child [7–14]. The relative contribution of maternal primary CMV infection during pregnancy to the risk of transmission to the fetus and newborn has been estimated at 40% (range 25 to 50%) for proven maternal primary CMV infections during pregnancy, and 1–2% for maternal non-primary or recurrent CMV infections during pregnancy. In addition, primary maternal infections are more often associated with symptomatic congenital CMV infection, and long term sequelae, including hearing loss [7].

It was previously thought that non-primary maternal CMV infections during pregnancy had both low transmission rates to the fetus and very low rates of symptomatic cCMV infection in the newborn, with a few exceptions found in isolated case reports. However, a growing body of evidence has indicated that maternal pre-conception immunity to CMV, while conferring some protection, is imperfect, and that maternal non-primary CMV infections during pregnancy may result in both asymptomatic and symptomatic cCMV, even with severe manifestations, in a much larger percentage of newborns than previously thought [15–28]. Still other studies have suggested that the age, race/ethnicity, and other demographics of the maternal population may influence the rates of maternal CMV infection (primary vs non-primary), rates of CMV transmission to the fetus and newborn, the presence of symptoms at birth in the newborn, and the outcomes in the children [29, 30].

In this study we examine the relationship of maternal primary vs. non-primary CMV infection during pregnancy to long-term sensorineural hearing loss outcomes in children born with congenital CMV infection, and compare the relationship to uninfected control subjects. This information has important implications for CMV vaccine trial development, as well as for identifying target populations for CMV vaccine administration once a licensed CMV vaccine is available for prevention of congenital CMV infection.

## Methods

### Study subjects

The Houston Congenital CMV Longitudinal Study began in 1981 and enrolled subjects through 2005. It is a prospective, longitudinal, multidisciplinary, natural history study of cCMV now in its 38^th^ year. The original hypothesis was that both symptomatic and asymptomatic congenital CMV infection in the newborn was associated with long-term sequelae in vision, hearing, and development in the child and adolescent, and that these outcomes could be predicted by type of maternal CMV infection in pregnancy and by newborn characteristics at birth [2, 3, 31–33].

**Fig 1:**
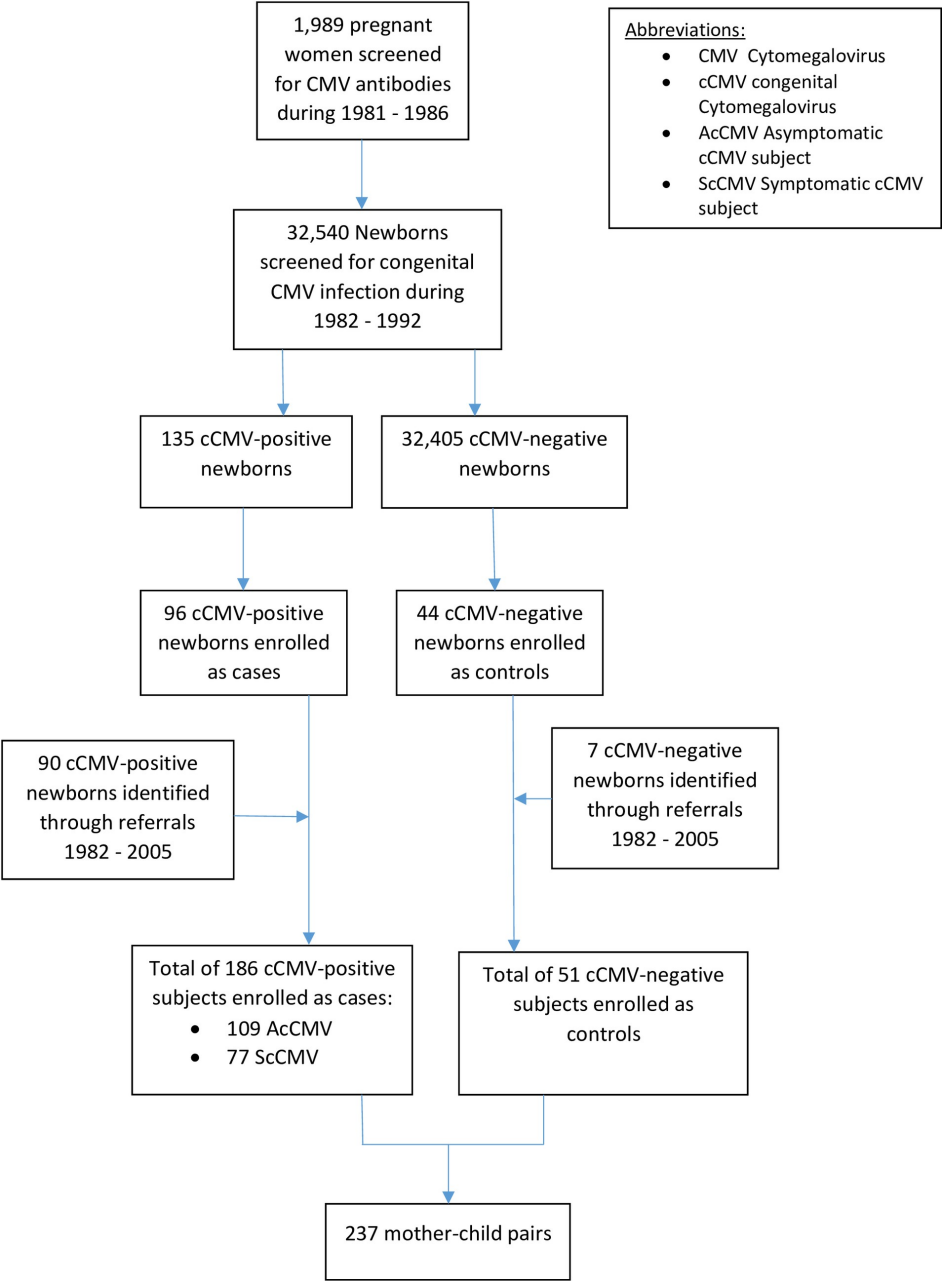
The Houston longitudinal CMV study.

In a sub-study of CMV infection in mothers and their infants, during 1981 to 1986, 4,578 pregnant women who were also patients of a private obstetric group practice participating in The Houston Congenital CMV Longitudinal Study were enrolled and screened prospectively for CMV IgM and IgG antibodies, beginning at the first obstetrical visit, after providing informed consent. The demographics and seroprevalence results of the serologic screening have been previously published [34]. In addition, from 1986 to 1992, mothers with identified CMV infections during pregnancy who were referred to us from other obstetrical practices were also included in this study [31]. The newborns of both groups of mothers were screened for congenital CMV infection within 3 days of life during 1982–1992. Inclusion and exclusion criteria for enrollment of the cCMV-infected infants into the final study were being a normal healthy, term or near-term newborn, without neonatal complications that could confound outcomes, and obtaining informed consent from the mother willing to participate in an up-to 18-year-long followup study. The inclusion criteria for uninfected control infants were that they were matched to cases by gender, gestation age, and date of birth within 3 days; and obtaining informed consent from the mother the same as with case subjects. This resulted in a final study population of 109 asymptomatic and 77 symptomatic cCMV infants, 51 uninfected infants as controls, and their mothers, resulting in 237 mother-infant pairs (**Fig 1**).

This study was approved by the Baylor College of Medicine Institutional Review Board and all subjects were enrolled after obtaining written informed consent.

### Definitions for maternal CMV infections

Maternal sera for determination of CMV IgG and IgM antibodies were obtained as early in pregnancy as possible, and repeated at each trimester and at delivery when possible [34]. Definitions for the type of maternal CMV infection were adapted from prospective serologic and demographic studies of CMV infection in pregnant women that were previously published and contemporary to the time of the serologic screening process for this study [31]. The type of maternal infection for all study subjects (symptomatic, asymptomatic and controls) was classified as follows:

Primary maternal infection with CMV was defined as either seroconversion from CMV IgG negative antibody to CMV IgG positive antibody (proven primary maternal CMV infection) or the presence of both CMV IgG antibody and CMV IgM antibody during pregnancy (presumptive primary maternal CMV infection). The presumptive primary CMV infections were considered presumptive because CMV IgM antibody may last up to 3 to 6 months; therefore, some of these maternal CMV infections may have occurred in the months immediately prior to conception. In addition, mothers who were CMV IgG antibody negative during the first trimester of pregnancy, and did not have subsequent serologic testing, yet gave birth to a congenitally infected newborn, were also considered to have a proven primary maternal CMV infection. Both proven primary and presumptive primary maternal CMV infections were analyzed together as primary maternal CMV infections.

Recurrent, or non-primary, maternal CMV infection was defined as the presence of CMV IgG antibody before pregnancy, or the presence of CMV IgG antibody and the absence of CMV IgM antibody during the first trimester of pregnancy. Recurrent infections during pregnancy may be from reactivation of the mother’s endogenous strain or strains/genotypes of CMV (in latently infected mothers) or reinfection with a new strain or strains/genotypes of CMV, but it is not possible with present serologic techniques to determine which type of recurrent maternal CMV infection resulted in the birth of a congenitally infected newborn. Hence these infections were categorized as just non-primary.

Uninfected maternal status was defined as mothers who were CMV IgG seronegative in the first trimester and remained CMV IgG seronegative throughout pregnancy, and who delivered an uninfected newborn without congenital CMV infection. These mothers were categorized as having never been infected with CMV.

Unknown type of maternal CMV infection during pregnancy was defined as the presence of CMV IgG antibody and the absence of CMV IgM antibody in the mother at delivery. This classification, which was assigned for some symptomatic, asymptomatic and control subjects, could have been applied to mothers who would have had either an unverified primary CMV infection or an unverified non-primary infection during pregnancy, or (for mothers of control subjects only), unverified non-infection. Included in this category were mothers in whom no or incomplete serologic data were available.

### Serologic and virologic methods

CMV IgG antibody was determined by enzyme-linked immunosorbent assay and anticomplement immunofluorescence [34]. CMV IgM antibody was determined by enzyme linked immunosorbent assay and radioimmunoassay, as previously described [33]. The use of CMV IgG avidity testing to assess maturation of the CMV IgG response or interferon gamma release assays to measure cell mediated immunity to CMV, or PCR assays to measure CMV viremia levels in the blood were not available at the time of this study when the women were evaluated for their CMV infection status during pregnancy [35–38]. In addition, molecular genotyping was not performed because it was not available at the time of the study, so it was not possible to determine whether or not non-primary maternal CMV infections were a result of reactivation or re-infection [39].

Urine from newborns was collected by externally applied urine collection bag, usually obtained in the first 3 days of life, and always confirmed again with a second urine sample obtained before the first 21 days of life, and cultured for CMV using traditional cell culture techniques of human foreskin fibroblast (HFF) monolayers, and examined daily for 21 days for evidence of cytopathic effect characteristic for CMV [40]. Isolation of CMV in HFF cell culture in urine obtained in the first 21 days of life defined the presence of a congenital CMV infection in the newborn, and was the virologic standard at the time. The use of contemporary molecular assays for detection of CMV DNA in urine or saliva of newborns were in development at the time of this study, but were not routinely available at the time of this study. The methods used for maternal immunoglobulin detection, and for CMV detection in the urine of newborns with the human foreskin fibroblast assay, were consistent throughout the study period.

### Assessment and classification of newborns

Infants were examined at birth by their private pediatricians at the birthing hospital, and by one of the authors (GJDH) subsequently, and determined to be symptomatic at birth or asymptomatic at birth. Confirmed symptomatic cCMV disease was defined as a newborn with CMV detected by culture of urine samples collected within 3 weeks of life, who presented with at least one of the following classic CMV-related signs at birth: purpura/petechiae, jaundice, hepatosplenomegaly, microcephaly, unexplained neurological abnormality, elevated liver enzymes (alanine aminotransferase >100 IU), hyperbilirubinemia (total bilirubin >3mg/dl), hemolytic anemia, or thrombocytopenia (platelet count <75, 000/mm^3^). The presence of hearing loss alone at birth was not part of the defining condition of symptomatic cCMV disease for this study [33].

Confirmed asymptomatic cCMV infection was defined as a newborn with CMV detected by culture of urine samples collected within 3 weeks of life, who had a normal newborn examination, i.e., none of the symptoms defining symptomatic cases. During the time of the study, universal newborn hearing screening was not routine, so the presence or absence of hearing loss at birth was not part of the defining condition of asymptomatic cCMV infection for this study. Newborns whose urine did not grow CMV in cell culture were identified as being not congenitally infected with CMV and were identified as controls.

### Longitudinal follow-up evaluations

The infants were then examined at regular intervals at 1 month, 4, 6, 9, 12 and 18 months and at up to 11 intervals thereafter up to 18 years of age, by one of the authors (GJDH) and the multidisciplinary members of the Houston Congenital CMV Longitudinal Study Team, for growth, hearing, vision, neurodevelopmental milestones and cognitive assessments [2, 3, 33]. This study focuses on the hearing outcomes in these newborns as they relate to maternal CMV serologic status during pregnancy. Hearing evaluations included click and tone-burst auditory brainstem response (ABR), behavioral audiometry from 0.25 to 8 kHz, and tympanometry. Sensorineural hearing loss (SNHL) was defined as at least ≥25 dB hearing level (“mild” loss according to ASHA) for the click ABR or at any frequency for the corrected tone-burst or pure-tone air conduction results, in the absence of middle ear disorder, and normal tympanometry. Also, SNHL for each ear was categorized as congenital/early-onset when detected in the first ABR assessment at age ≤12 months and confirmed in subsequent assessments, or as delayed-onset when detected after one or more assessments with normal hearing; and categorized by laterality and severity, described elsewhere [3].

## Statistical analysis

### Maternal demographics

Maternal demographic information for age, race/ethnicity, parity, and gestational age at delivery were collected and described. Maternal CMV infection type (primary/presumptive primary, non-primary, no infection, or unknown) and category of newborn by birth symptoms (symptomatic, asymptomatic or control) were tabulated and described. The proportions and confidence intervals of children with SNHL up to 18 years of age were analyzed according to maternal CMV infection category. The primary and presumptive primary mothers were analyzed as one category.

Sensorineural hearing loss in relation to the timing of CMV infection in pregnancy was also examined. SNHL rates by trimester of infection (when this could be determined) was tabulated for symptomatic and asymptomatic cases. The presence of SNHL by first or second half of pregnancy was also tabulated and examined for significance with a chi-square test.

Socioeconomic status of the mothers was determined using a formula that took into account various economic indicators based on the zip code of residence at birth from the US Census Bureau and the presence or absence of private insurance assessed from patient records. This methodology allows the assessment of various components of SES at the neighborhood level such as income ranges, housing characteristics, employment and poverty, and has been used to associate SES with health outcomes, disease risk and health disparities [41–51].

### Survival analysis

#### A. Life tables

Survival analysis methods employed Life Table analysis [52–54]. This method analyzes time-to-event and survival of censored subjects that were grouped into intervals of the first year of life, second and third years, 3-year intervals thereafter up to age 15, and 15 through 28 years. Patients experiencing hearing loss or who were censored in the previous interval are not carried forward into person-years at risk for detectable hearing loss in the subsequent interval. For each time interval, the probability density (the probability of hearing loss divided by the person-years at risk during the interval), the hazard rate and the cumulative proportion surviving hearing loss-free at the end of the interval were computed.

Separate Life Tables were computed for maternal primary infection, non-primary infection, uninfected, and unknown maternal infection categories of patients. For primary vs. non-primary maternal infection groups, relative risks were computed for proportions with hearing loss, probability densities, and hazard rates in the first year of life. Life Table analysis excluding Control (uninfected) subjects was also done.

#### B. Kaplan-Meier

For a more granular look at hearing loss survival, the Kaplan-Meier Product Limit Method was used to take into account the exact times of documented hearing loss and censorship in symptomatic cCMV and asymptomatic cCMV cases in the four maternal infection categories rather than aggregating these into time intervals [48]. Only symptomatic and asymptomatic cCMV cases were used with this method in order to examine hearing loss survival among those who were actually congenitally infected, thus eliminating the dilution of hearing loss risk in the cohort posed by the uninfected controls, who had a low risk of hearing loss. Kaplan-Meier survival function graphs were constructed incorporating the four maternal infection categories (primary, non-primary, uninfected, and unknown) showing hearing loss and censorship events. Pairwise statistical comparisons were made with the Wilcoxon statistic and significance testing. The survival distributions are compared overall and pairwise using the log-rank test. Because the hazard rates for the four maternal infection groups may not be the same throughout the follow-up period, two variants of the log-rank test were also employed: the Generalized Wilcoxon (Gehan) and the Tarone-Ware tests, which give more weight to hearing loss events earlier in the follow-up period and accommodate censored patients.

Mean age at censoring was compared across the three maternal infection categories with Analysis of Variance; equality of mean censorship times was further assessed with Welch and Brown-Forsythe statistics, and pairwise comparisons were made with t-tests. Survival curves for symptomatic, asymptomatic and control subjects were also generated and compared as above.

### Simulating complete ascertainment of maternal infection type

Since the type of maternal infection was not completely ascertained on all mothers, but symptom classification at birth (symptomatic, asymptomatic or control) and hearing loss status were known for all children, simulations were undertaken to further estimate the influence of maternal CMV infection type on hearing outcomes. These were done separately in symptomatic and asymptomatic groups because of suspicions that most cCMV infections among symptomatic patients result from primary maternal infection, but among asymptomatic patients this assumption is less clear.

Because the maternal CMV infections in the unknown category were likely to be a mix of both primary and non-primary maternal CMV infections, simulations were conducted to reassign symptomatic and asymptomatic newborns with unknown type of maternal infection into existing primary or non-primary maternal infection categories in various ratios. The hearing loss rates in these simulated, pooled maternal infection categories was then reassessed after augmentation with the reassigned children. Controls were not used in the simulations. The rate of hearing loss in the simulated, pooled primary maternal infection category then resulted from the existing rate of hearing loss in children known to have primary maternal infection combined with the hearing loss rate in those who were reassigned from unknown to primary maternal infection. Similarly, the hearing loss rate in the simulated, pooled non-primary infection category was determined from the hearing loss rate in those already known to have non-primary infection, combined with the hearing loss rate in those reassigned from unknown to non-primary infection, assuming those with unknown type of maternal infection took their hearing loss rate with them when they were reassigned. The new hearing loss rate in each simulated maternal infection category was determined by the number with hearing loss divided by the new denominator population.

Kaplan-Meier curves were generated for hearing loss and censorship events in these simulated, pooled maternal infection categories. Survival distributions, including those of symptomatic cases before and after the simulated reassignment, were compared similarly to the above.

Age distributions in subjects from primary and unknown maternal CMV infection categories were compared with the Kolmogorov-Smirnov test.

## Results

### Maternal demographics

Demographic data showed most of the mothers were white, of non-Hispanic ethnicity, married, with a medium to high socioeconomic status (SES), who delivered term or near-term newborns. Their mean age overall was 27 years. The mean age of the mothers who delivered newborns with symptomatic cCMV infection were younger than those mothers who delivered newborns with asymptomatic cCMV (23.8 years vs 28.5 years, P<0.001). Symptomatic newborns were more likely to be from the referral group rather than the screened group, and more likely delivered to younger mothers of lower SES status **(Table 1)**.

**Table 1:** Demographic birth characteristics of mothers of infants enrolled in the Houston congenital CMV longitudinal study, by type of maternal CMV infection during pregnancy.

	Primary or PP	non-Primary	Unknown	Uninfected	Totals
Symptom Type:					
Symptomatic	12 (16.4)	4 (14.8)	61 (48.0)		77 (32.5)
Asymptomatic	51 (69.9)	18 (66.7)	40 (31.5)		109 (46.0)
Control	10 (13.7)	5 (18.5)	26 (20.5)	10 (100.0)	51 (21.5)
Total	73	27	127	10	237
Mother’s Race & Ethnicity:					
White Hispanic	5 (6.8)	1 (3.7)	25 (19.7)	0 (0.0)	31 (13.1)
White Non-Hisp	62 (84.9)	22 (81.5)	78 (61.4)	10 (100.0)	172 (72.6)
Black Non-Hisp	6 (8.2)	4 (14.8)	22 (17.3)	0 (0.0)	32 (13.5)
Other	0 (0.0)	0 (0.0)	2 (1.6)	0 (0.0)	2 (0.8)
Marital Status:					
Unmarried	3 (4.1)	5 (18.5)	28 (22.0)	0 (0.0)	36 (15.2)
Married	66 (90.4)	20 (74.1)	96 (75.6)	10 (100.0)	192 (81.0)
Unknown	4 (3.4)	2 (7.4)	3 (2.3)	0 (0.0)	9 (3.8)
Mother’s Age:					
Under 18	3 (4.1)	2 (7.4)	12 (9.4)	0 (0.0)	17 (7.2)
18–25	22 (30.1)	8 (29.6)	46 (36.2)	1 (10.0)	77 (32.5)
26+	48 (65.8)	15 (55.6)	68 (53.5)	7 (70.0)	138 (58.2)
Unknown	0 (0.0)	2 (7.4)	1 (0.7)	2 (20.0)	5 (2.1)
Mean Age, y (sd)	28.0 (5.3)	28.2 (5.3)	26.0 (6.1)	29.4 (3.4)	27.0 (6.0)
Gender of Child:					
Male	40 (54.8)	13 (48.1)	69 (54.3)	8 (80.0)	130 (54.9)
Female	33 (45.2)	14 (51.9)	58 (45.7)	2 (20.0)	107 (45.1)
Previous Pregnancies:					
Zero	17 (23.3)	9 (33.3)	48 (37.8)	3 (30.0)	77 (32.5)
One	25 (34.2)	11 (40.7)	37 (29.1)	1 (10.0)	74 (31.2)
Two	20 (27.4)	3 (11.1)	19 (15.0)	2 (20.0)	44 (18.6)
Three	5 (6.8)	1 (3.7)	10 (7.9)	0 (0.0)	16 (6.8)
4 or more	6 (8.2)	0 (0.0)	5 (3.9)	0 (0.0)	11 (4.6)
Unknown	0 (0.0)	3 (11.1)	8 (6.3)	4 (40.0)	15 (6.3)
Mean no. of pregnancies (sd)	1.45 (1.25)	0.83 (0.82)	1.08 (1.24)	0.83 (0.98)	1.17 (1.21)
No. of Living Children:					
Zero	24 (32.9)	12 (44.4)	63 (49.6)	4 (40.0)	103 (43.5)
1	34 (46.6)	10 (37.0)	39 (30.7)	2 (20.0)	85 (35.9)
2	11 (15.1)	2 (7.4)	14 (11.0)	0 (0.0)	27 (11.4)
3 or more	4 (5.5)	0 (0.0)	3 (2.4)	0 (0.0)	7 (2.9)
unknown	0 (0.0)	3 (11.1)	8 (6.3)	4 (40.0)	15 (6.3)
Mean number of living children (sd)	0.93 (0.84)	0.58 (0.65)	0.65 (0.82)	0.33 (0.52)	0.73 (0.81)
Socio-Economic Status (from Zip Code + Insurance)					
Low	3 (4.1)	5 (18.5)	30 (23.6)	0 (0.0)	38 (16.0)
Medium	20 (27.4)	7 (25.9)	41 (32.3)	2 (20.0)	70 (29.5)
High	50 (68.5)	15 (55.6)	56 (44.1)	8 (80.0)	129 (54.4)
From Screened Population					
No	16 (21.9)	5 (18.5)	76 (59.8_	2 (20.0)	99 (41.8)
Yes	57 (78.1)	22 (81.5)	51 (40.2)	8 (80.0	138 (58.2)
Gestational Age					
< 34 weeks	3 (4.1)	0 (0.0)	7 (5.5)	0 (0.0)	10 (4.2)
34 to 36 6/7	8 (11.0)	3 (11.1)	23 (18.1)	0 (0.0)	34 (14.3)
37 to 38 6/7	17 (23.3)	8 (29.6)	35 (27.6)	1 (10.0)	61 (25.7)
39+ weeks	45 (61.6)	15 (55.6)	57 (44.9)	4 (40.0)	12 (51.1)
Unknown	0 (0.0)	1 (3.7)	5 (3.2)	5 (50.0)	11 (4.6)

### Maternal infection type and newborn symptom classification

Both primary and non-primary maternal infections resulted in symptomatic and asymptomatic cCMV in the newborns. Of the 77 newborns with symptomatic cCMV infection, 12 (16%) of mothers had proven or presumed primary CMV infection during pregnancy, 4 (5%) had known non-primary CMV infection during pregnancy, and 61 (79%) were categorized as having unknown type of maternal CMV infection. Of the 109 newborns with asymptomatic cCMV infection, 51 (47%) of mothers had proven or presumed primary CMV infection during pregnancy, 18 (17%) had known non-primary CMV infection during pregnancy, and 40 (37%) were categorized as unknown type of maternal CMV infection. Of the 51 uninfected newborn controls, 10 (20%) of mothers had proven or presumptive primary CMV infection during pregnancy, five (10%) had non-primary CMV infection during pregnancy, 10 (20%) were never infected with CMV during pregnancy, and 26 (51%) were categorized as unknown type of maternal CMV infection **(Table 2)**.

**Table 2:** Symptom status and sensorineural hearing loss by maternal CMV infection category.

	Primary	non-Primary	Uninfected	Unknown	Totals
Symptomatic	9/12 (75%)	0/4 (0%)	-	48/61 (79%)	57/77 (74%)
Asymptomatic	14/51 (27)	2/18 (11)	-	6/40 (15)	22/109 (20)
Control	1/10 (10)	0/5 (0)	1/10 (10)	1/26 (4)	3/51 (6)
**Totals n/N (%)**	24/73 (33)	2/27 (7)	1/10 (10)	55/127 (56)	82/237 (35)
Duration of Followup for SNHL, y	17.5 (8.2)^A^	15.2 (10.3)	19.6 (4.5)	13.9 (8.1)	**15.4 (8.5)**
Mean (SD)					
Age When SNHL First Detected, y	4.6 (6.0)^B^	0.5 (0.4)	15 (n/a)	1.2 (2.5)	2.4 (4.3)
Mean (SD)					
Congenital HL, n/N (%)	11/24 (46)^C^	2/2 (100)	0/1 (0)	42/55 (76)	55/237 (23)
Delayed-onset HL, n/N (%)	13/24 (54)^C^	0/2 (0)	1/1 (100)	13/55 (24)	27/237 (11)
Maximum Degree of Hearing Loss (ASHA classification)	4 Mild*	2 Profound	1 Moderate	22 Mild	26 Mild
	5 Moderate*			3 Moderate	9 Moderate
	2 Severe			2 Severe	4 Severe
	13 Profound			28 Profound	43 Profound

^A^ Significantly different from the unknown category at p < .001 by student t-test.

^B^ Significantly different from the unknown category at p = .018 by student t-test.

^C^ Significantly different from unknown category at p = .008 by chi-square test.

* Significantly different from the unknown maternal infection category at p < .05 by z-test.

### Maternal infection type, timing, newborn category, and sensorineural hearing loss

Of the 77 symptomatic newborns, 57 (74%) had hearing loss by 18 years of age. Of the symptomatic newborns whose maternal infection type could be determined, 9 out of 12 (75%) born to mothers with primary maternal CMV infection had hearing loss versus zero out of 4 with non-primary maternal CMV infection (p = .009). Of the 61 symptomatic patients with unknown type of maternal infection, 48 (79%) had hearing loss **(Table 3)**.

**Table 3:** Proportions of newborns with congenital CMV infection with and without hearing loss by age 18 years of age, and type of maternal CMV infection.

	Symptomatic			Asymptomatic			Control			Row Totals		
Hearing Loss (HL)	HL/*n*	Proportion (95% CI)	*p*	HL/*n*	Proportion (95% CI)	*p*	HL/*n*	Proportion (95% CI)	*p*	HL/*n*	Proportion (95% CI)	*p*
**Primary**	9/12	75.0%	**.009**	14/51	27.5%	.157	1/10	10.0%	.462	24/73	32.9%	**< .001**
								(0.0–28.6)			(22.1–43.7)	
		(50.5–99.5)			(15.2–39.7)							
**non-Primary**	0/4	0		2/18	11.1%		0/5	0		2/27	7.4%	
					(0.0–25.6)						(0.0–17.3)	
**No Infection**	-	-		-	-		1/10	10.0%		1/10	10.0%	
											(0.0–28.6)	
								(0.0–28.6)				
**Unknown**	48/61	78.7%		6/40	15%		1/26	3.8%		55/127	43.3%	
		(68.4–89.0)			(3.9–26.1)			(0.0–11.2)			(34.7–51.9)	
**Column Totals**	57/77	74.0%		22/109	20.2%		3/51	5.9%		82/237	34.6%	
								(0.0–12.3)			(28.5–40.7)	
		(64.2–83.8)			(12.6–27.7)							
**Trimester of Infection (Primary Infections Only):**												
1^st^	2/3	66.7%		3/9	33.0%		0/4	0.0%		5/16	31.3%	
		(13.3–1.00)			(2.5–64.1)						(8.5–54.0)	
1^st^ or 2^nd^	0/0	0.0%		1 / 2	50%		0/0	0%		1/2	50.0%	
					(0.0–1.0)							
											(0.0–1.0)	
2^nd^	1/1	100%		3/11	27.3%		0/0	0%		4/12	33.3%	
		(1.0–1.0)			(0.1–53.6)						(6.7–60.0)	
2^nd^ or 3^rd^	2/2	100%		2/6	33.3%		0/0	0%		4/8	50.0%	
											(15.4–84.6)	
		(1.0–1.0)			(0.0–71.1)							
3rd	3/3	100%		5/20	25.0%		1/5	20.0%		9/28	32.1%	
					(6.0–44.0)			(0.0–55.1)			(14.8–49.4)	
		(1.0–1.0)										
Unknown	1/3	33.3%		0/3	0.0%		0/1	0.0%		1/7	14.3%	
		(0.0–86.7)									(0.0–40.2)	
Totals	9/12	75%		14/51	27.5%		1/10	10.0%		24/73	32.9%	
					(15.2–39.7)			(0.0–28.6)			(22.1–43.7)	
		(50.5–99.5)										
**Pregnancy Half When Infected (Primary Infections Only)**												
**First Half of Pregnancy**	3/4	75%	.444	5/15	33.3%	.669	0/4	0.0%	1.000	8/23	34.8%	.858
		(32.6–1.0)			(9.5–57.2)						(15.3–54.2)	
**Second Half of Pregnancy**	5/5	100%		9/33	27.3%		1/5	20.0%		14/43	32.6%	
		(1.0–1.0)			(12.1–42.5)			(0.0–55.1)			(18.6–46.6)	
**Unknown**	1/3	33.3%		0/3	0%		0/1	0%		1/7	14.3%	
		(0.0–86.7)									(0.0–40.2)	
**Totals**	8/12	75.0%		14/51	27.5%		1/10	10.0%		23/73	31.5%	
		(40.0–93.3)									(20.9–42.2)	
					(15.2–39.7)			(0.0–28.6)				

Among the 109 asymptomatic patients, 22 (20%) developed hearing loss. Of the 51 asymptomatic newborns born to mothers with known primary maternal CMV infection, 14 (28%) developed hearing loss, versus 2 out of18 (11%) of asymptomatics with non-primary infection (p = .157). There were 40 asymptomatic newborns born to mothers with unknown type of maternal infection, six (15%) of whom had hearing loss **(Table 3)**.

Among the 51 controls, three (6%) developed hearing loss: one born from the 10 mothers with primary CMV infection versus 0/5 of those were born to mothers with non-primary CMV infection (p = .462)); one was born from the 10 mothers confirmed to have no maternal CMV infection; and one born from the 26 mothers with unknown maternal CMV infection type **(Table 3)**.

There was no significant relationship identified for the timing of primary maternal infection in relation to sensorineural hearing loss in the child. There were no significant differences in the proportions of subjects with sensorineural hearing loss in those born to mothers who acquired primary infections in the first half vs. the second half of pregnancy. This held true for symptomatic, asymptomatic and control subjects born to mothers with primary infections **(Table 3)**.

### Survival analysis of hearing loss

#### A. Life tables

**Fig 2:**
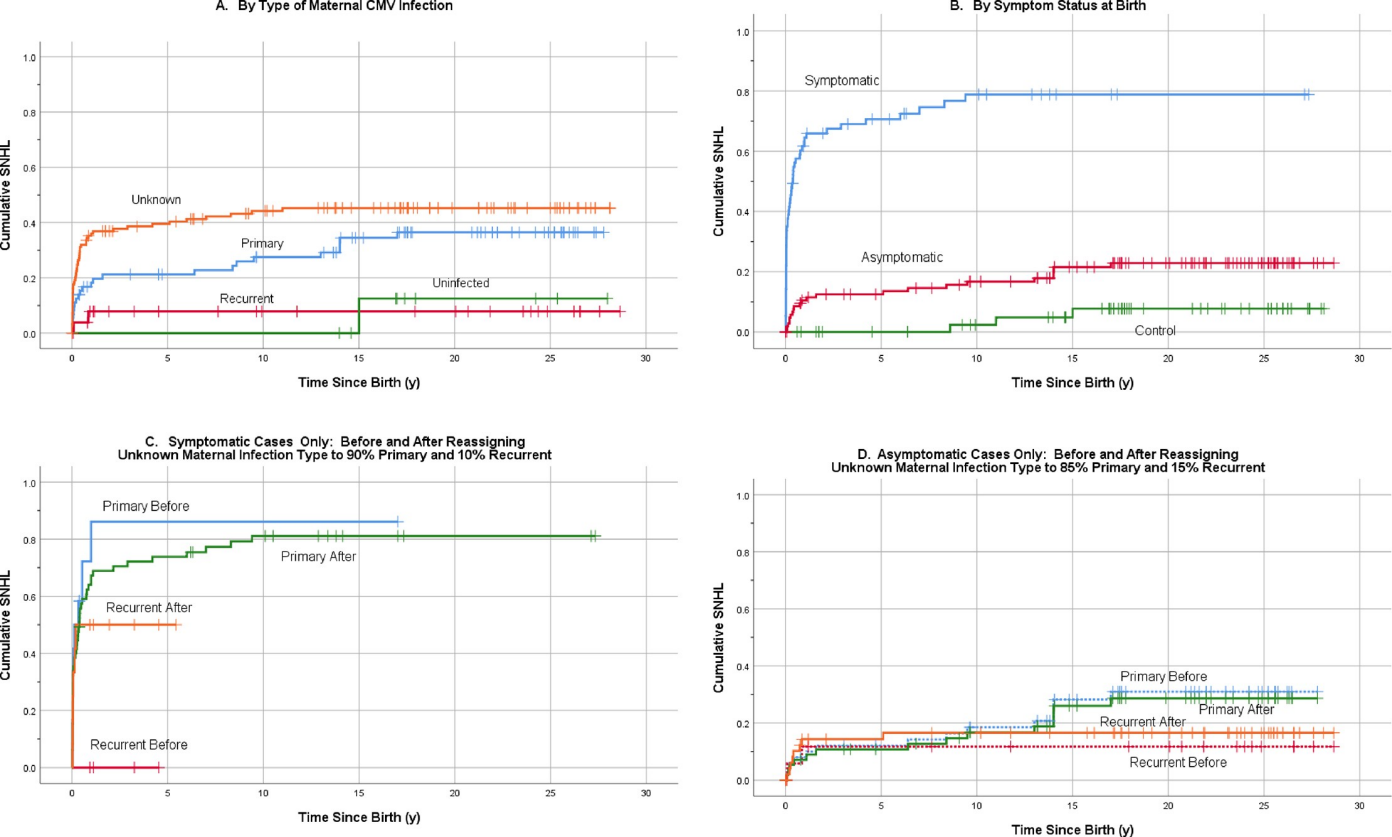
Kaplan-Meier hearing loss survival curves for maternal infection categories. (A) Survival curves by maternal serology type. Comparison of Survival Curves by Log-Rank test: Unknown vs. primary (p = .068); Unknown vs. non-Primary (p = .001); Unknown vs. uninfected (p = .042); Primary vs. non-Primary (p = .026). (B) Survival curves by symptom group at birth. Comparison of Survival Curves by Log-Rank test: Symptomatic vs. Asymptomatic (p < .001); Symptomatic vs. Control (p < .001); Asymptomatic vs. Control (p = .019). (C) Survival curves of symptomatic cases before and after reassigning those with unknown type of maternal CMV infection into Primary and Recurrent infection categories. Comparison of Survival Curves by Log-Rank test: Primary Before vs. Primary After (p = .576); Non-Primary Before vs. non-Primary After (p = .107); Primary Before vs. non-Primary Before (p = .016); Primary After vs. non-Primary After (p = .313). (D) Survival curves for asymptomatic cases before and after reassigning those with unknown type of maternal CMV infection into Primary and Recurrent (non-Primary) infection categories. Primary Before vs. Primary After (p = .797), Non-Primary Before vs. non-Primary After (p = .651), Primary Before vs. non-Primary Before (p = .218), Primary After vs. non-Primary After (p = .333).

Life tables for hearing loss were computed for each maternal CMV infection type. These tables show that most hearing loss events occurred in the first year of life. Of the ten uninfected control subjects who were born to mothers known to have no evidence of CMV infection during pregnancy (the “no infection” category), and who all had periodic assessments for hearing loss, one developed significant hearing loss at age 15 years. This subject was a known hunter with environmental exposure to gunshots, and did not consistently use ear protection. This is not characteristic of a typical pattern of congenital CMV-associated hearing loss. This one subject provides strong evidence that very late-onset hearing loss may not always be associated with, or caused by, congenital CMV infection, but rather more likely by environmental exposures such as firearms or loud music (**Tables 2 and 4, Fig 2**).

**Table 4:** Life tables for hearing loss in the child according to type of maternal CMV infection during pregnancy.

	LIFE TABLE FOR HEARING LOSS											
	A. For Primary/Presumed Primary Maternal Infection											
Interval Start Time	Number Entering Interval	Number Withdrawing during Interval	Number Exposed to Risk	Number of Terminal Events	Proportion Terminating	Proportion Surviving	Cumulative Proportion Surviving at End of Interval	Std. Error of Cum. Proportion Surviving at End of Interval	Probability Density	Std. Error of Probability Density	Hazard Rate	Std. Error of Hazard Rate
0	73	5	70.500	12	.17	.83	.83	.04	.170	.045	.19	.05
1	56	0	56.000	3	.05	.95	.79	.05	.044	.025	.06	.03
2	53	0	53.000	0	.00	1.00	.79	.05	.000	.000	.00	.00
3	53	3	51.500	0	.00	1.00	.79	.05	.000	.000	.00	.00
6	50	0	50.000	3	.06	.94	.74	.05	.016	.009	.02	.01
9	47	2	46.000	1	.02	.98	.72	.05	.005	.005	.01	.01
12	44	6	41.000	4	.10	.90	.65	.06	.023	.011	.03	.02
15	34	9	29.500	1	.03	.97	.63	.06	.007	.007	.01	.01
18	24	1	23.500	0	.00	1.00	.63	.06	.000	.000	.00	.00
21	23	7	19.500	0	.00	1.00	.63	.06	.000	.000	.00	.00
24	16	13	9.500	0	.00	1.00	.63	.06	.000	.000	.00	.00
27	3	3	1.500	0	.00	1.00	.63	.06	.000	.000	.00	.00
	B. For non-Primary Maternal Infection											
Interval Start Time	Number Entering Interval	Number Withdrawing during Interval	Number Exposed to Risk	Number of Terminal Events	Proportion Terminating	Proportion Surviving	Cumulative Proportion Surviving at End of Interval	Std. Error of Cum. Proportion Surviving at End of Interval	Probability Density	Std. Error of Probability Density	Hazard Rate	Std. Error of Hazard Rate
0	27	3	25.500	2	.08	.92	.92	.05	.078	.053	.08	.06
1	22	3	20.500	0	.00	1.00	.92	.05	.000	.000	.00	.00
2	19	0	19.000	0	.00	1.00	.92	.05	.000	.000	.00	.00
3	19	2	18.000	0	.00	1.00	.92	.05	.000	.000	.00	.00
6	17	1	16.500	0	.00	1.00	.92	.05	.000	.000	.00	.00
9	16	3	14.500	0	.00	1.00	.92	.05	.000	.000	.00	.00
12	13	0	13.000	0	.00	1.00	.92	.05	.000	.000	.00	.00
15	13	1	12.500	0	.00	1.00	.92	.05	.000	.000	.00	.00
18	12	2	11.000	0	.00	1.00	.92	.05	.000	.000	.00	.00
21	10	3	8.500	0	.00	1.00	.92	.05	.000	.000	.00	.00
24	7	5	4.500	0	.00	1.00	.92	.05	.000	.000	.00	.00
27	2	2	1.000	0	.00	1.00	.92	.05	.000	.000	.00	.00
	C. For No Infection Category											
Interval Start Time	Number Entering Interval	Number Withdrawing during Interval	Number Exposed to Risk	Number of Terminal Events	Proportion Terminating	Proportion Surviving	Cumulative Proportion Surviving at End of Interval	Std. Error of Cum. Proportion Surviving at End of Interval	Probability Density	Std. Error of Probability Density	Hazard Rate	Std. Error of Hazard Rate
0	10	0	10.000	0	.00	1.00	1.00	.00	.000	.000	.00	.00
1	10	0	10.000	0	.00	1.00	1.00	.00	.000	.000	.00	.00
2	10	0	10.000	0	.00	1.00	1.00	.00	.000	.000	.00	.00
3	10	0	10.000	0	.00	1.00	1.00	.00	.000	.000	.00	.00
6	10	0	10.000	0	.00	1.00	1.00	.00	.000	.000	.00	.00
9	10	0	10.000	0	.00	1.00	1.00	.00	.000	.000	.00	.00
12	10	2	9.000	0	.00	1.00	1.00	.00	.000	.000	.00	.00
15	8	4	6.000	1	.17	.83	.83	.15	.056	.051	.06	.06
18	3	0	3.000	0	.00	1.00	.83	.15	.000	.000	.00	.00
21	3	0	3.000	0	.00	1.00	.83	.15	.000	.000	.00	.00
24	3	2	2.000	0	.00	1.00	.83	.15	.000	.000	.00	.00
27	1	1	.500	0	.00	1.00	.83	.15	.000	.000	.00	.00
	D. For Unknown Maternal Infection Category											
Interval Start Time	Number Entering Interval	Number Withdrawing during Interval	Number Exposed to Risk	Number of Terminal Events	Proportion Terminating	Proportion Surviving	Cumulative Proportion Surviving at End of Interval	Std. Error of Cum. Proportion Surviving at End of Interval	Probability Density	Std. Error of Probability Density	Hazard Rate	Std. Error of Hazard Rate
0	127	4	125.000	44	.35	.65	.65	.04	.352	.043	.43	.06
1	79	4	77.000	2	.03	.97	.63	.04	.017	.012	.03	.02
2	73	1	72.500	2	.03	.97	.61	.04	.017	.012	.03	.02
3	70	2	69.000	2	.03	.97	.60	.04	.006	.004	.01	.01
6	66	4	64.000	3	.05	.95	.57	.05	.009	.005	.02	.01
9	59	5	56.500	2	.04	.96	.55	.05	.007	.005	.01	.01
12	52	8	48.000	0	.00	1.00	.55	.05	.000	.000	.00	.00
15	44	13	37.500	0	.00	1.00	.55	.05	.000	.000	.00	.00
18	31	5	28.500	0	.00	1.00	.55	.05	.000	.000	.00	.00
21	26	11	20.500	0	.00	1.00	.55	.05	.000	.000	.00	.00
24	15	10	10.000	0	.00	1.00	.55	.05	.000	.000	.00	.00
27	5	5	2.500	0	.00	1.00	.55	.05	.000	.000	.00	.00

#### B. Kaplan-Meier survival analysis

Graphs generated from Kaplan-Meier survival analysis show that subjects with cCMV who were born to a mother with a non-primary maternal CMV infection had a relatively lower cumulative proportion of hearing loss over the age span, and the onset of hearing loss in this group was identified either at birth or within the first year of life. Subjects born with cCMV as a result of primary maternal CMV infection had a relatively higher cumulative proportion of hearing loss, with the hearing loss in this group either identified at birth or continuing to accrue up to 17 years of age. Subjects with cCMV born to mothers with an unknown-type maternal CMV infection had an even higher proportion of hearing loss at birth and in the first year of life, and continued to accrue hearing loss up to 11 years of age. The survival distributions were significantly different between primary and non-primary maternal CMV infection (p = .026), and non-primary and unknown maternal CMV infection (p = .001) by log-rank tests. There was no significant difference in survival distributions between primary and unknown maternal CMV infection (p = .068) by log-rank tests (**Fig 2**). However, the mean age at first hearing loss onset was significantly different between primary and unknown maternal CMV infection categories (p = .001 by student t-test) **(Table 5)**.

**Table 5:** Age at censorship or first sensorineural hearing loss, by maternal infection serotype.

Age at Censorship (No Hearing Loss)									Age at Hearing Loss
Maternal Infection Type	N	Mean	Std. Deviation	Std. Error	95% Confidence Interval for the mean		Minimum	Maximum	Mean (SD) When SNHL Detected
					Lower Bound	Upper Bound			
Primary	49	17.6	8.5	1.2	15.1	20.1	.00	27.8	4.6 (6.0)
non-Primary	25	14.6	10.7	2.1	10.2	19.0	.06	28.6	0.5 (0.4)
No Infection	9	19.5	5.0	1.7	15.6	23.4	14.0	28.0	15.0
Unknown	72	16.2	8.4	1.0	14.2	18.2	.00	28.1	1.2 (2.5)
**Totals**	155	16.6	8.7	0.7	15.2	18.0	.00	28.6	2.4 (4.3)

### Censored subjects

Subjects who did not develop known hearing loss were censored on average 14.2 years after the average age of first hearing loss onset among those who did develop hearing loss (p< .001). It is therefore highly unlikely there was significant undetected or hidden hearing loss among the censored study subjects. Many subjects who withdrew (censored cases) did so after age 15 years, but most hearing loss occurred well before that age **(Table 5)**.

### Estimated hearing loss outcomes after simulated ascertainment of maternal CMV infection type

Subjects with mothers who were in the unknown maternal infection category were reassigned to either primary or non-primary infection categories in simulations.

In the first simulation, involving only the symptomatic at birth category of subjects, 90 percent of those with unknown type of maternal infection were reassigned into primary maternal infection and 10 percent reassigned into the non-primary maternal infection category. This 90/10 reassignment into the known maternal infection categories was chosen based on current estimates from epidemiologic studies, which suggest that 90% of symptomatic cCMV cases result from primary maternal infection. This simulation was also reformulated to reassign 80/20, 70/30, and 60/40 of unknowns to primary and non-primary maternal infection, respectively, for symptomatic subjects.

In simulations with the asymptomatic at birth category of subjects, reassignments 60/40, 50/50, 40/60, and 30/70 were made to maternal primary and non-primary infection categories, respectively **(Table 6)**.

**Table 6:** Simulated reassignment of symptomatic and asymptomatic subjects with unknown type of maternal infection to primary or recurrent maternal infection categories, and the resulting percentages of sensorineural hearing loss.

Maternal Infection Category	Currently Known Hearing Loss/Total (%)		‘Unknown’ Serotype		Resulting Estimated Proportion with Hearing Loss	
			Proportion Reassigned to Category	No. Added to Category:	Point Estimate	95% Confidence Interval
				*n* with HL /		
				*N* Reassigned		
**SYMPTOMATIC:** 79% of subjects with ‘Unknown’ maternal infection type have hearing loss					
Currently Known:					
Primary/PP	9/12 (75%)	0	0	75.0%	(50.5–99.5%)
non-Primary	0/4 (0%)	0	0	0	-
Unknown	48/61 (79%)			78.7%	(68.4–89.0%)
Primary/PP	9/12 (75%)	.90	43/55	52/67 (77.6%)	(67.6–87.6%)
non-Primary	0/4 (0%)	.10	5/6	5/10 (50%)	(19.0–81.0%)
Primary/PP	9/12 (75%)	.80	39/49	48/61 (78.7%)	(68.4% - 89.0%)
non-Primary	0/4 (0%)	.20	9/12	9/16 (56.3%)	(31.9–80.6%)
Primary/PP	9/12 (75%)	.70	34/43	43/55 (78.2%)	(67.3–89.1%)
non-Primary	0/4 (0%)	.30	14/18	14/22 (63.6%)	(43.5–83.7%)
Primary/PP	9/12 (75%)	.60	29/37	38/49 (77.6%)	(65.9–89.2%)
non-Primary	0/4 (0%)	.40	19/24	19/28 (67.9%)	(51.0–85.1%)
**ASYMPTOMATIC:** 15% of subjects with ‘Unknown’ serology type have hearing loss					
Currently Known:					
Primary/PP	14/51 (27%)	0	0	27.5%	(15.2–39.7%)
non-Primary	2/18 (11%)	0	0	11.1%	(0.0–25.6%)
Unknown	6/40 (15%)			15.0%	(3.9–26.1%)
Primary/PP	14/51 (27%)	.30	2/12	16/63 (25.4%)	(14.4–35.6%)
non-Primary	2/18 (11%)	.70	4/28	6/46 (13.0%)	(3.3–22.8%)
Primary/PP	14/51 (27%)	.40	2/16	16/67 (23.9%)	(13.7–34.1%)
non-Primary	2/18 (11%)	.60	4/24	6/42 (14.3%)	(3.7–24.9%)
Primary/PP	14/51 (27%)	.50	3/20	17/71 (23.9%)	(14.0–33.9%)
non-Primary	2/18 (11%)	.50	3/20	5/38 (13.2%)	(2.4–23.9%)
Primary/PP	14/51 (27%)	.60	4/24	18/75 (24.0%)	(14.3–33.7%)
non-Primary	2/18 (11%)	.40	2/16	4/34 (11.8%)	(0.9–22.6%)

For symptomatic subjects, after random reassignment of children with unknown type of maternal infection to primary/non-primary categories in ratios of 90/10, 80/20, 70/30 and 60/40, the point estimates for the proportion of children with hearing loss ranged from 77.6% to 78.7% in the simulated primary maternal infection group. Among these symptomatic children, the hearing loss rates in the simulated primary infection groups was similar to the rate for those with known primary infection before simulation with the augmented cases, regardless of reassignment ratio. This is due to the similar rates of hearing loss in the primary (75%) and unknown (79%) groups for symptomatic children before the reassignments.

For symptomatic subjects in the simulated non-primary infection category in the above ratios, 50.0% to 67.9% had hearing loss. The estimated rate of hearing loss was higher than in those with known non-primary maternal infection before the simulation, owing to the fact that so few symptomatic patients were ascertained to have a known non-primary infection, none of whom had hearing loss, and to the high rate of hearing loss among those with unknown type of maternal infection who were reassigned into this non-primary category. For these symptomatic subjects, as the proportion of those reassigned from unknown to non-primary maternal CMV infection got higher in the simulations, the rate of hearing loss in the simulated non-primary category increased **(Table 6)**.

For asymptomatic subjects, the estimated point estimates for hearing loss in the simulated groups after augmentation with those of unknown maternal infection type ranged from 23.9% to 25.4% in the simulated primary maternal infection group, and from 11.8% to 14.3% in the simulated non-primary maternal infection group. These estimated rates of hearing loss did not differ much across the different reassignment ratios and did not greatly differ from the hearing loss rates in the known primary or known non-primary maternal CMV infection categories for asymptomatic subjects before the reassignments **(Table 6)**.

Results of these simulations show that if the maternal CMV infection status of subjects was fully ascertained, hearing loss rates among symptomatic subjects born to mothers with primary maternal infection would likely not meaningfully change from our actual observed rate of 75%, regardless of the ratio of primary/non-primary maternal infection. However, higher hearing loss rates would be expected among symptomatic subjects born to mothers with non-primary maternal CMV infection, compared to the 0/4 or 0% rate of hearing loss that was documented in symptomatic subjects who were known to have been born to mothers with non-primary maternal CMV infection in this study. Again this is due to the low number of symptomatic subjects who were known to have maternal non-primary infections, and the high (79%) rate of hearing loss in the symptomatic subjects with unknown maternal infection type.

These results also suggest that among asymptomatic subjects, full ascertainment of maternal infection category would not likely meaningfully change the known hearing loss rates that were actually observed in asymptomatic subjects with known primary or known non-primary maternal CMV infection.

To further elucidate the results of simulated reassignments of those with unknown maternal serology type to either primary or non-primary maternal infection, Kaplan-Meier graphs were generated. For the symptomatic subjects, we graphed a random reassignment of 90% of subjects born to mothers with unknown maternal serology type to primary maternal infection category, and 10% to the non-primary maternal infection category, where they were combined with those already known to be in those categories as described above. The actual hearing loss status and dates of onset for the known and randomly-reassigned patients were used. The graphs show the hearing loss survival distributions of subjects born to mothers with known primary and non-primary maternal infection categories, before any reassignments were made, and after the addition of reassigned unknowns to these categories (Fig 2).

Results show that, in the symptomatic group, there is no statistical difference between hearing loss survival distributions of the known primary maternal infection group before reassignments were made and after reassignments of unknowns to this category (p = .576 by Log-Rank test). The distributions also show no difference between the known non-primary maternal infection group before reassignments and after reassignments of unknowns to that group. All three variants of the log-rank test show no significant differences between the before and after groups (**Fig 2**). The similarity of hearing loss survival distributions, before and after reassignments, supports the assumption that many or most of the symptomatic subjects born to mothers with unknown maternal CMV infection type, may result from primary maternal CMV infections.

The survival distributions for the 109 asymptomatic subjects also show no statistical differences between hearing loss survival distributions before and after simulated reassignments, for either primary or non-primary type of maternal CMV infection (**Fig 2**). In addition, separate analysis of only those 92 asymptomatic at birth subjects identified through the hospital-based newborn screening program showed no statistical differences in hearing loss survival distributions before and after simulated reassignments, for either primary or non-primary maternal CMV infections.

## Discussion

Both primary and non-primary maternal infections resulted in symptomatic cCMV infection, usually indicating a more severe outcome of cCMV infection. Both primary and non-primary maternal CMV infections also resulted in asymptomatic cCMV infection. Symptomatic cCMV infection was more likely to occur after primary maternal CMV infection in pregnancy. Younger maternal age appeared to be an independent risk factor for delivery of a baby with symptomatic cCMV infection while the type of infection (primary or non-primary) was controlled for.

Hearing loss occurred in subjects born to mothers with primary or non-primary CMV infections, and in subjects with cCMV infection who were asymptomatic at birth or symptomatic at birth. Hearing loss was more common in subjects born with symptomatic cCMV compared to asymptomatic cCMV infection. In addition, most hearing loss occurred in children born with cCMV infection as a result of maternal primary CMV infection than non-primary maternal CMV infections (in both symptomatic and asymptomatic subjects), although the timing of maternal primary infection in pregnancy appeared to have no effect on the rates of sensorineural hearing loss. In all subjects with cCMV infection, hearing loss was common and usually occurred at birth or within the first year of life. The latest age of onset of hearing loss observed was 17 years of age. Kaplan-Meier survival curves also showed a higher proportion of onset of hearing loss in subjects over time born to mothers with primary CMV infection, as well as those with an unknown type of maternal CMV infection during pregnancy, compared to those born to mothers with a known non-primary CMV infection, although the number of mothers ascertained to have known non-primary maternal CMV infection was small.

In the uninfected controls, hearing loss also occurred, but at a later time in childhood, at a much lower rate, and exhibited a different pattern than in the subjects with cCMV infection. This comparative baseline of hearing loss in uninfected children, compared to congenitally infected children, may be useful in planning a vaccine trial to prevent maternal CMV infection with a trial endpoint of reducing hearing loss in children and adolescents. Our findings suggest that a CMV vaccine trial would not require an extended followup time in order to capture most cCMV-related hearing loss. It also shows that study dropouts after the early followup period would not obscure significant undocumented hearing loss in controls.

Among symptomatic children, the hearing loss rate of those born to mothers with an unknown type of maternal CMV infection was similar to the rate in in those born to mothers with a known primary CMV infection. And in simulations where children born to mothers of unknown type of CMV infection were reassigned to the symptomatic group with known maternal primary infections, the rate of hearing loss in the augmented symptomatic group was not significantly different from before the simulated reassignments. This similarity lends support to the belief that symptomatic congenital CMV infection is usually the result of primary maternal CMV infection, which confers a higher risk of hearing loss than non-primary maternal CMV infection.

Only a small number of mothers were ascertained to have known non-primary CMV infection and gave birth to children categorized in the symptomatic cCMV category at birth (n = 4), none of whom developed hearing loss. However, the simulated reassignments of symptomatic children with unknown type of maternal infection into the non-primary maternal infection category show that non-primary maternal infections would likely confer a considerable hearing loss rate as well. This finding suggests children born with symptomatic cCMV infection, as a result of non-primary maternal CMV infection during pregnancy, are at a relatively higher risk for hearing loss than was previously appreciated for those with non-primary maternal infection.

Subjects categorized in the asymptomatic group at birth and born to mothers with known non-primary type of maternal CMV infection (n = 18), also had a considerable 11% (95% CI = 0–25%) known hearing loss rate, and after augmentation with the simulated reassignments of subjects with unknown maternal serology category to this category, the hearing loss rates for this category were the same or slightly increased.

It is likely that most of the unknown types of maternal CMV infections in this study were actually primary CMV infections, especially for the children born with symptomatic cCMV infection.

Our results suggest that prevention of both primary and non-primary maternal CMV infection during pregnancy is likely to significantly reduce the incidence of hearing loss in children.

Life table analysis may be helpful in planning trials to evaluate the effect of an intervention to prevent cCMV-associated hearing loss in children. CMV is a dynamic infectious agent with multiple transmission vectors whose timing and impact on cCMV incidence rates are not fully understood. It is therefore not clear which vectors of CMV transmission are the most important for maintaining incident cCMV in a population and could be disrupted by vaccination, e.g., toddler-to-toddler, toddler-to-mother, adult-to-mother-to-fetus, etc. Life table analysis is especially useful in this regard to determine the incidence and prevalence of cCMV-related hearing loss in various age groups and would allow the computation of age-adjusted hearing loss rates. It allows the determination of the probability density (the probability of hearing loss divided by the person-years at risk in the time interval) for different age groups or subpopulations, and to compare hearing loss hazard ratios between these groups. These comparisons could be made within a cohort or between entire cohorts that do and do not receive a CMV vaccine. Life table analysis would be especially informative in community trials of a CMV vaccine, for example where community cohorts of subjects may be enrolled all at once to establish the ongoing incidence and prevalence of cCMV and its related hearing loss before and after a vaccine is introduced. In a community setting, incident cases of CMV-related hearing loss in one-time interval or age group would diminish the number of persons at-risk for CMV-related hearing loss in subsequent time intervals; and conferred immunity to CMV in one age group might decrease incident CMV infection rates in younger or older age groups going forward. Even for an imperfect CMV vaccine, life table analysis can aid in determining the optimal targets, age groups and timing for vaccination that would be needed to disrupt transmission or significantly mitigate hearing loss outcomes. It also allows the delayed effects of immunity, waning immunity, and herd immunity to be observed in a cohort, depending on the study design. It is informative to determine baseline cCMV-related hearing loss rates in different age strata, as our study has done, before CMV vaccine trials are initiated. Although our life table methodology might have been more amenable to a larger community cohort study of cCMV, it did show from a natural history perspective that most cCMV-related hearing loss occurred in the first year of life or by three years of age at a time when few if any prenatal or public health messages about cCMV were extant and no CMV vaccine yet exists.

This study is the first to document the relative role of maternal primary vs maternal non-primary CMV infection in hearing loss in children born with asymptomatic cCMV infection, and to relate the long-term outcome of hearing loss through adolescence to maternal serologic status. Previous studies have focused on symptomatic newborns, but asymptomatic newborns, because they represent the more common manifestation of cCMV, likely constitute the greater public health burden from cCMV. Prevention of both primary and non-primary types of maternal CMV infection, and transmission to the fetus, may therefore reduce the public health impact of cCMV infection.

This study has limitations. Although diversity is represented in our study, this population provides information on primarily white, non-Hispanic, middle to upper SES mothers, in Texas, and may not be generalizable to all maternal populations or geographic areas.

Another limitation is a lack of definitive ascertainment of maternal CMV serologic status for many of the subjects enrolled. This limitation was minimized through simulated reassignments of subjects born to mothers with unknown type of maternal CMV infection into primary or non-primary categories in biologically plausible ratios, and the determination of the resulting hearing loss rates. Furthermore, since the women were identified and tested serologically for CMV infection over 30 years ago, contemporary methods, including CMV IgG avidity, interferon gamma release tests, and molecular nucleic acid detection methods to determine CMV reactivations versus reinfections in the non-primary CMV infection category, were not available. However, since most practicing obstetricians will use only the standard CMV IgG and CMV IgM antibody tests to screen or diagnose pregnant women for CMV infection, the results from this study still have great clinical significance.

The results from this study have relevance for CMV vaccine development [55]. Progress towards a successful CMV vaccine to prevent cCMV infection or ameliorate the effects of cCMV disease has been hampered by a lack of understanding of all the correlates of protective immunity [18, 19]. Maternal CMV IgG seropositive status does not appear to prevent non-primary maternal CMV infections and transmission to the fetus and newborn, nor does it appear to prevent long-term sequelae of sensorineural hearing loss. Therefore clinical trials of vaccine safety and efficacy will need to take into consideration this “imperfect” natural immunity measured by CMV IgG serum antibody in the pregnant woman, and consider the role of other maternal immune or epigenetic factors, as well as fetal and placental factors. Based on the currently evolving knowledge about the relative role of primary vs non-primary CMV infection during pregnancy, a successful CMV vaccine must be able to not only prevent primary CMV infection and transmission to the fetus, but also boost natural immunity to reduce transmission to the fetus from non-primary CMV infections. Understanding the relative role of reactivation vs reinfection in maternal non-primary CMV infections may help vaccine development as well. Ultimately, health policies will have to consider the most efficacious methods of CMV vaccination, taking into account these considerations.

While development of a CMV vaccine continues, prevention of both primary and non-primary CMV infections in pregnancy remains a challenge. Studies have shown prenatal behavioral interventions, consisting of CMV awareness education about common behaviors associated with CMV transmission to pregnant women, are effective in changing behaviors and may reduce transmission of CMV to pregnant women [56]. Because CMV infection and disease as well as long-term sequelae may occur after both primary and non-primary maternal infections, these behavioral interventions should be recommended for all pregnant women, regardless of CMV serostatus.

Since cCMV infection occurs after both maternal primary and non-primary CMV infections, identification of newborns with cCMV infection through universal newborn screening is likely to be the best way to detect cCMV infection in all newborns, both symptomatic and asymptomatic at birth, born to mothers with both primary and non-primary CMV infections [57, 58]. Early identification of these newborns would allow the proper anticipatory guidance for detecting long-term sequelae, such as hearing loss and neurodevelopmental disabilities, as well as provide appropriate antiviral therapies for those newborns who may benefit from such therapy [59].
